# Dependence on the socio-economic system impairs the sustainability of pasture-based animal agriculture

**DOI:** 10.1038/s41598-023-41524-4

**Published:** 2023-08-31

**Authors:** Enrique Muñoz-Ulecia, Alberto Bernués, Andrei Briones-Hidrovo, Isabel Casasús, Daniel Martín-Collado

**Affiliations:** 1 Department of Animal Science, Agrifood Research and Technology Centre of Aragon (CITA), Avda. Montañana 930, 50059 Zaragoza, Spain; 2 AgriFood Institute of Aragon – IA2 (CITA-University of Zaragoza), Zaragoza, Spain; 3 Research Centre for Energy Resources and Consumption (CIRCE), University of Zaragoza-Campus Río Ebro, 50018 Zaragoza, Spain

**Keywords:** Environmental sciences, Environmental social sciences, Energy science and technology

## Abstract

Livestock systems contribution to environmental change is controversial. Pasture-based systems are considered a sustainable alternative due to their adaptation to the use of local natural resources. However, they have limited productivity per product unit and, in Europe, depend on public economic support. Furthermore, they are heterogeneous in farm structure and resources use, which may determine their sustainability. We use emergy accounting to assess the sustainability of mountain pasture-based cattle systems and analyse the variability among farms. Emergy accounting assesses the sustainability performance of complex systems (i.e., farming systems) and their interaction with other systems (i.e., the environment and the socio-economic system) focusing on the origin, quality and quantity of the energy required for the system to function. Results show that pasture-based systems largely use local natural renewable resources but depend largely on the wider socio-economic system given their reliance on public economic support and purchased animal feeds. This economic dependence turns out in most farms largely using non-renewable resources. Increasing self-produced feeds and grazing on natural pastures can reduce the dependence on the socio-economic system and improve farm sustainability.

## Introduction

Agriculture is not only a major driving force for trespassing planetary boundaries, such as biodiversity loss, biogeochemical flows disruption and climate change intensification^1,2^, but also one of the most important drivers of ecosystem services degradation^3^. Of all agriculture forms, livestock systems lie at the core of the public and scientific debate because of their controversial contribution to environmental change^4,5^, resulting in a marked focus to assess their sustainability in the academy. Nonetheless, livestock farming systems are widely diverse in production orientation, farming practices and use of resources, and provide contrasting social, economic and environmental outcomes^6,7^. Consequently, sustainability assessments should differentiate among farming systems to better understand the specific social and environmental role of livestock on the global and local scale^6,8^.

Life cycle assessment (LCA) is the most widely used approach to assess farming systems’ environmental sustainability^9^. The outcomes of these assessments are strongly influential because they are used to inform policies and decision making^10^, which is reflected, for instance, in the European Union GHG reduction goals^11^. However, LCA does not consider the contribution of free renewable resources (such as solar radiation or rain), how much it takes for the geobiosphere to produce required inputs (e.g., indirect resources and energy), or the energy and resources associated to economic exchanges^12^. Consequently, from the LCA perspective, conventional intensive farming systems, which are highly efficient in the use of resources and emissions per product unit, are normally considered as having a low environmental impact. However, not considering the issues mentioned above leaves LCA failing to fully address whether farming activity can be maintained in the long term^13,14^.

From a systemic perspective, pasture-based systems are usually singled out as a sustainable alternative for keeping livestock farming within planetary boundaries, while maintaining ecosystem functions and services^15^ due to their adaptation to local environments, considerable use of natural resources and self-sufficiency^16,17^. However, these systems normally depend on public economic support, particularly in the European Union^18^. In addition, they have limited feed conversion efficiency and productivity per product unit^19,20^, commonly resulting in low sustainability performance when applying LCA approaches. Moreover, pasture-based systems are usually characterised for their heterogeneity in terms of both farm structure and local resources use^18,21,22^, which can lead to variable sustainability performance. Thus, considering farm diversity is crucial for the sustainability assessments of these systems to be sound and accurate.

One of the alternative approaches to assess livestock systems’ sustainability is to focus on the energy required for their functioning and maintenance. This energy comes from renewable (solar radiation flux) and non-renewable (fossil fuel stocks) sources. Considering the origin, quantity and quality of used resources is essential for assessing long-term sustainability. From this perspective, emergy accounting assesses the available energy that has been required to generate a product or service after considering all the direct and indirect energy embodied in resources, as well as the different qualities of the energy used in the production process^13,23^. Within the energy framework, energy quality refers to the ability of different energy types to do useful work, where useful can be defined as contributing to the preservation of the system^13,24^.

In the last two decades, several studies have used emergy accounting to compare farming systems managements (usually conventional vs. pasture-based, organic, or low-input/low-output farming systems)^25–29^. In these emergy studies, conventional intensive livestock systems generally present lower environmental sustainability than less intensive managements. In the case of beef cattle farming, the few emergy studies published are based on data from single farms^30–34^, and/or average data from national or regional databases^35–38^, and do not address heterogeneity among farms. Very little is known about how individual farms may present variable sustainability outcomes depending on their own particularities.

The objectives of this study were to: (i) assess the sustainability of mountain pasture-based beef-cattle systems, understood as the capacity to maintain their activity over time based on the resources used and the load placed on the environment, using emergy accounting; and (ii) analyse the variability of sustainability performance in terms of the energy used among individual farms. We discuss the implications of the results for selecting farming practices that have potential to improve farms’ sustainability.

## Material and methods

### Study area and data collection

The study area comprised three valleys from the Spanish Central Pyrenees, which had been previously selected to embrace the diversity of mountain beef-cattle management practices and environmental and socio-economic contexts^18,39–41^. Data about farm structure, farming management and economic performance were collected from farms (n = 50) by means of an in-depth face-to-face questionnaire in 2018. These farms are part of a longitudinal study and have been followed up in 1990, 2004 and 2018. The research protocol and questionnaire content, and all methods were performed in accordance with the guidelines and with the approval of the Ethics Committee of the Agrifood Research and Technology Centre of Aragón, Spain (no. CESIH_2022_3). Data anonymity was granted to the participants, who expressed their oral informed consent to provide the information contained in the questionnaire.

There were two farm types according to the production objectives. Most farms sold weaned calves to be fattened elsewhere (weaner farms; n = 40), and the rest fattened calves on-farm, which implied more animal feed purchases (mainly concentrates), and they sold animals ready for slaughter (weaner-finisher farms; n = 10). The studied farms represented around one fourth of all the cattle farms in these valleys in 2018 (official data from the Aragón Statistics Institute). The main structural and economic characteristics of the studied farms are presented in Table 1 and is further detailed in^18^.

**Table 1:** Structural and economic characteristics of the studied farms. Mean and SD.

Variable	Variable definition	Weaner farms (n = 40)		Weaner-finisher farms (n = 10)	
		Mean	SD	Mean	SD
Agricultural area (ha)	Sum of private area used for crops, forages, pastures, and other agricultural uses. This area does not include public/communal grazing areas	47.3	31.0	67.6	52.2
Herd size (LU)	Livestock units of cattle, where the coefficient used was: 1 for cows and bulls; 0.7 for heifers; 0.4 for calves	72.8	33.8	123.0	73.1
Labour input (WU)	One work unit (WU) is equivalent to the work of one person, full time, for one year	1.3	0.5	1.7	0.9
Grazing length (days)	Days of grazing without external feeds input	251.5	49.4	237.5	24.0
Total income (€)	The sum of incomes obtained from the sale of farm products	41,548	28,004	82,952	45,508
CAP Payments (€)	Payments for agriculture maintenance and development	32,420	16,473	56,112	37,570
Variable costs (€)	Feeding costs plus veterinary costs, water and electricity, transport, fertilizers and miscellaneous items	23,722	13,609	56,691	45,063
Gross margin (€)	Total income plus subsidies, minus variable costs	50,247	32,268	82,373	44,301
Gross margin/LU (€)	Gross margin per livestock unit	717.6	395.4	705.0	148.0

Grazing management in the area is a longstanding tradition^17^, characterised by using different natural resources in spring, summer and autumn. Animals are moved annually from (rented or owned) private meadows and forest areas in the valleys where farms are located, where they graze in spring, to communal mountain pastures in summer before being moved back to the valleys in autumn^42^. This grazing management lasts two thirds of the year^18^ and is a major source of livestock feeding^43^. In winter, animals are mostly kept in farm surrounding areas and have access to barns. Most farms complete the herd diet (in winter and while grazing in valleys) with self-produced forage crops and purchased straw, corn, forage and concentrates. Mountain communal pastures are regulated and entail paying fees to use them. Farms are supported by public policies (the first and second pillars of the Common Agricultural Policy, CAP). These CAP payments represent around two thirds of these farms’ gross margin^18^.

### Emergy framework

Emergy accounting method looks back on the production chain to consider the different upstream energy inputs of every energy type (e.g., fossil fuels, electricity or energy embedded in resources), which must be included to summarise all the energy required in any production process^13^. Emergy computes the difference in quality between the various energy forms involved in a process and expresses them all as the same unit (i.e., sej). To do so, all the system’s inputs (e.g., animal feeds, crop inputs, fuels, human labour, CAP payments) must be converted into emergy units with a conversion factor called the Unit Emergy Value (UEV), which is the emergy embedded in one unit of a specific product or service^13^. Therefore, the UEV represents the available energy that has been directly and indirectly required to produce a good divided by the total amount produced, considering all the processes and transformations that took place to produce it. With this conversion, emergy accounting allows comparisons of the renewable and non-renewable resources from the environment, as well as local and external resources from the socio-economic system^44^. Renewable energy is defined as the energy that directly or indirectly comes from natural renewable sources (i.e., sun radiation), while non-renewable energy is that which comes from fossil fuels or is used up faster than its renewal rate (i.e., soil erosion)^13^. The more energy transformations take place, the higher the UEV is because during each transformation, available energy is consumed to produce a smaller amount of energy of another form and some energy is dissipated^13,45^. The emergy methodological framework consists of three main steps:

(i) First, drawing of the diagram defining the system’s boundaries and the inputs, outputs, and flows of resources.
(ii) Second, compiling all the flows of the resources indicated in the diagram in an emergy evaluation table, where resources are converted into sej using the UEV. The UEV is defined depending on the resource type: the emergy to energy ratio (Transformity, for resources in energy units), emergy per mass (specific emergy, for resources in mass units) or the emergy that supports the generation of one economic product unit (em€, for resources in monetary units, e.g., CAP payments)^46^. The em€ represents how much emergy corresponds to a unit of money produced by the national economy^47^. UEVs have to be checked and homogenised according to the global emergy baseline (GEB). The GEB is the sum of the primary energies driving all the processes of the geobiosphere, commonly assessed on a yearly basis^48^. We applied the renewability factor to account for the renewable and non-renewable fraction of each resource^32^. This factor was obtained from the literature based on the proportion of renewable emergy required to produce the product or service under analysis. To avoid double counting of natural resources (i.e., solar radiation, wind, rain and evapotranspiration) of the same origin (i.e., solar radiation), only that with the highest emergy value must be used^13^. Finally, all the resource flows incorporated into the evaluation table have to be classified into four types, namely: natural renewable local resources (R), natural non-renewable local resources (N), purchased resources (P), and services (S). The emergy yield (Y) is calculated as the sum of the previous ones.
(iii) Third, calculation of emergy indicators (Table 2).

**Table 2:** Description and calculation of emergy indicators.

Emergy indicator	Definition	Formula
Renewability (%R)	The ratio between natural renewable local resources (R) and the total emergy of the system. Represents renewability	$\frac{R}{Y}$
Emergy Yield Ratio (EYR)	The ratio between the emergy yield (Y) and the emergy from purchased resources (P) and services (S). Represents net contribution to the socio-economic system	$\frac{Y}{P+S}$
Emergy Investment Ratio (EIR)	The ratio between the emergy from purchased resources (P) and services (S) and the emergy from natural local (renewable or not) resources (R and N). Represents market dependency	$\frac{P+S}{R+N}$
Emergy Exchange Ratio (EER)	The ratio between the emergy yield (Y) and the money paid for a product or service. Represents market trade status	$\frac{Y}{{{\EUR} \cdot \left[ {\frac{seJ}{{\EUR} }} \right]}}$
Environmental Loading Ratio (ELR)	The ratio between non-renewable natural (N) or purchased (P and S) emergy resources divided by natural renewable (R) ones. Represents environmental load	$\frac{N+P+S}{R}$
Emergy Sustainability Index (ESI)	The ratio between EYR and ELR. Represents sustainability	$\frac{\frac{Y}{{P}_{n}+{S}_{n}}}{\frac{N+{P}_{n}+{S}_{n}}{R+{P}_{r}+{S}_{r}}}$

P_r_ and S_r_ are the renewable fraction of the purchased resources and services, while P_n_ and S_n_ are the non-renewable fraction of the purchased resources and services. ^a^ To calculate the ESI, the alternative calculation of EYR and ELR proposed by^49^ has been used, which included the renewable and non-renewable fraction of each resource.

### Accounting for grazing resources from natural pastures in emergy accounting

The standard approach to assess emergy from natural pastures considers that they receive energy from natural renewable resources and that, if the area used by livestock is known, the emergy flow can be fully allocated to livestock grazing^29,31,32,37^. However, this approach is problematic because: (i) natural pastures are not on farmland, which generates the problem of properly accounting for their contribution to farms’ sustainability performance; (ii) livestock does not consume all the energy that natural pastures receive. Here we propose an alternative approach to estimate the emergy flow from natural pastures more accurately by considering grazing period length (days), the stocking rate and the proportion of Aboveground Net Primary Production (ANPP) in relation to Net Primary Production (NPP), and the ANPP consumed by livestock as follows:

1 $$Emergy\; natural \; pastures \left(sej\right)= E{mergy \; grazing}_{m\&f} \left(sej\right)+ E{mergy \; grazing}_{mp} (sej)$$

where _*m&f*_ refers to meadows and forests, _*mp*_ refers to mountain pastures, and *Emergy grazing* is the emergy that livestock obtains in each grazing area, calculated as:

2 $$Emergy \; grazing (sej) = \frac{E (sej/yr)}{grazing \; length \; (days/yr) \cdot ANPP (\%) \cdot A{NPP}_{consumed} (\%)}$$

where *E* is the emergy flow for all the NPP of these ecosystems calculated as described in Sect. "Emergy framework". using the stocking rate of 0.2 LU/ha^50^ to calculate the area in meadows and forests; 1.2 LU/ha^51^ in mountain pastures; *Grazing length* refers to the number of days that livestock are grazing; *ANPP* is 50% for both grazing areas^52,53^; *ANPP*_*consumed*_ is the proportion of ANPP consumed by livestock, estimated at 65% for meadows and forests and 40% for mountain pastures^52^.

### Emergy calculations

We followed the three steps described in Sect. "Emergy framework" for the 50 farms under study. We present the results separately for farms according to their productive orientation as weaner farms and weaner-finisher farms are not comparable because they have different outputs. Then we calculated emergy indicators individually for each farm as proxies of their sustainability performance in terms of the energy used. Services are assets or work that do not constitute a farm’s biophysical input or output but affect its economic performance. So they were included in the analysis as suggested^13,32^. Details about the calculations of each emergy flow are provided in the Supplementary material.

### System diagram and boundaries

**Figure 1:**
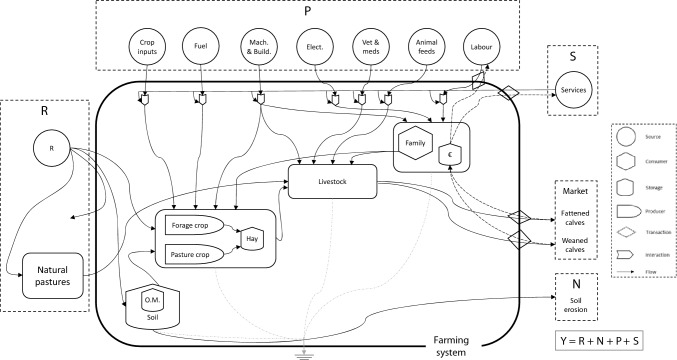
Emergy diagram representing the studied grazing livestock farming system. R: natural renewable local resources. N: natural non-renewable local resources. P: purchased resources. S: services. Y: emergy yield. O.M. refers to organic matter. Crop inputs includes seeds, fertilisers and phytochemicals. Mach. & Build. It includes machinery and buildings’ maintenance and depreciation, and small equipment. Animal feeds include straw, corn, forage, vitamin-mineral supplements and concentrates. Services include taxes paid and subsidies (CAP payments) received by farmers.

Figure 1 shows the emergy diagram that represents the studied farming system. On the one hand, farms received renewable energy from sun radiation, rain, wind and evapotranspiration, and also from natural pastures (i.e., R). On the other hand, farms interacted with the socio-economic system by purchasing resources (P; i.e., crop inputs, animal feeds and other farming inputs, while also hiring labour force), and exchanging services (S; i.e., paying taxes and receiving public economic support in the form of subsidies). The natural pastures-farms interaction consists in an inflow of biomass from natural pastures to farms, an outflow of organic matter from livestock to pastures, and economic exchange between farms and institutions for using natural pastures (not shown in the figure).

## Results

### Emergy flows—What resources contribute

**Figure 2:**
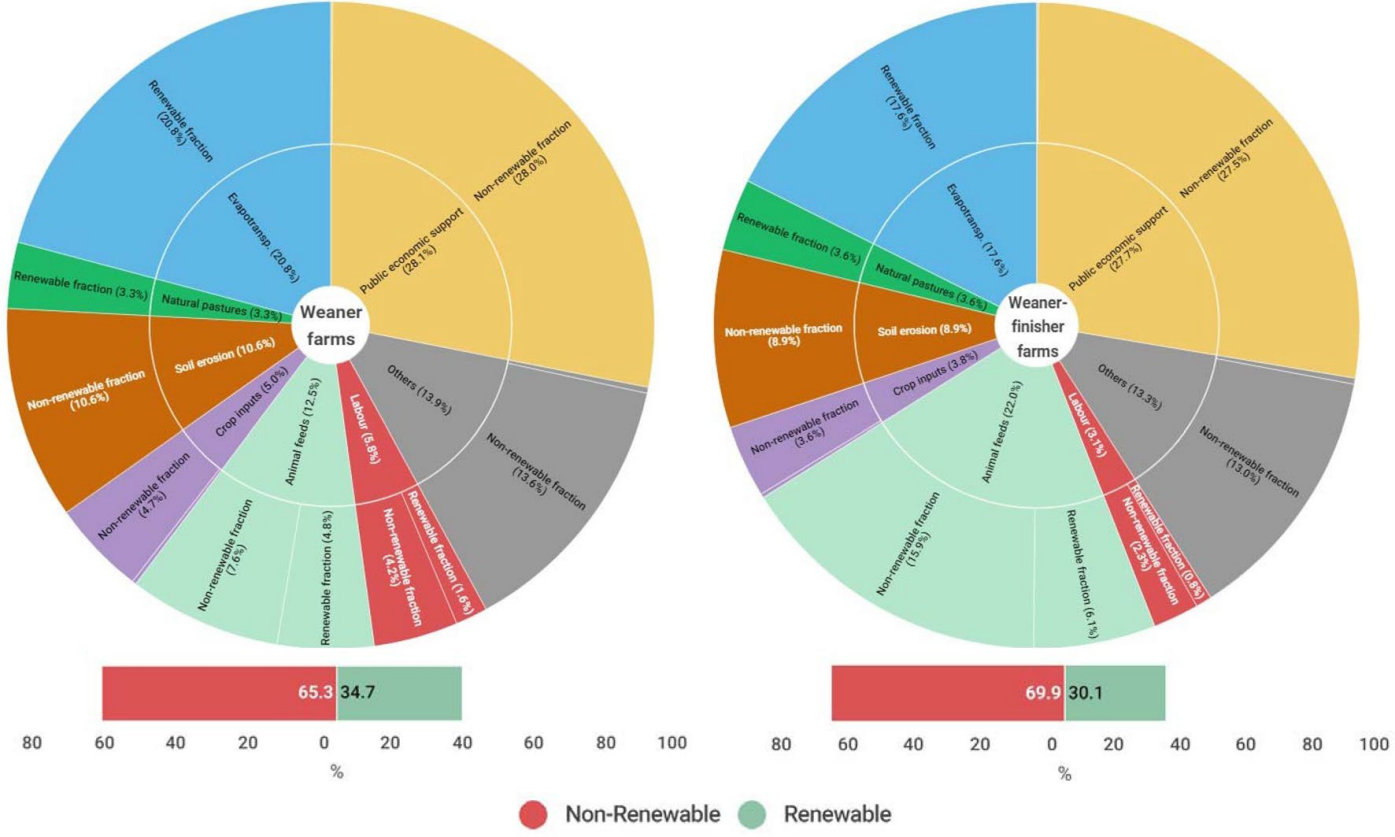
Average emergy flow for each group of resources by differentiating farming systems. Services include CAP payments received (28.7% and 28.1%, respectively for the weaner and weaner-finisher farms), minus taxes paid by farmers (0.6% and 0.4%). Others include machinery (2.5% and 2.9%), buildings (4.7% and 3.4%), small equipment (1.1% and 1.2%), veterinary and medicines (2.1% and 2.1%), electricity (0.1% and 0.1%) and fuel (3.4% and 3.7%). Animal feeds include straw (3.7% and 5.2%), forage (6.0% and 3.2%), vitamin-mineral supplements (0.2% and 0.3%) and concentrates (2.4% and 13.4%). Crop input includes seeds (0.0% and 0.0%), fertiliser (4.9% and 3.7%) and phytochemicals (0.0% and 0.0%). Each colour refers to a group of resources.

The average proportion of each resource in farms’ total emergy flow is provided in Fig. 2, differentiating the renewable and non-renewable fraction of each resource. For the weaner farms, natural local resources (evapotranspiration, natural pastures, soil erosion) represented an average of 34.7% of the total emergy flow, with 24.1% coming from renewable resources. The resources from the socio-economic system represented 65.3% of the total emergy flow, having services (i.e., CAP payments), animal feeds and others (i.e., veterinary and medicines, machinery and buildings, electricity and fuel) the highest contribution (54.5%) in the emergy flow. For the weaner-finisher farms, the flows from natural local resources represented 30.1% of the total emergy flow, with 21.2% coming from renewable resources. The resources from the socio-economic system represented 69.9% of the total emergy flow, with animal feeds, services (i.e., CAP payments), and others (i.e., veterinary and medicines, machinery and buildings, electricity and fuel) contributing more (63.0%).

**Figure 3:**
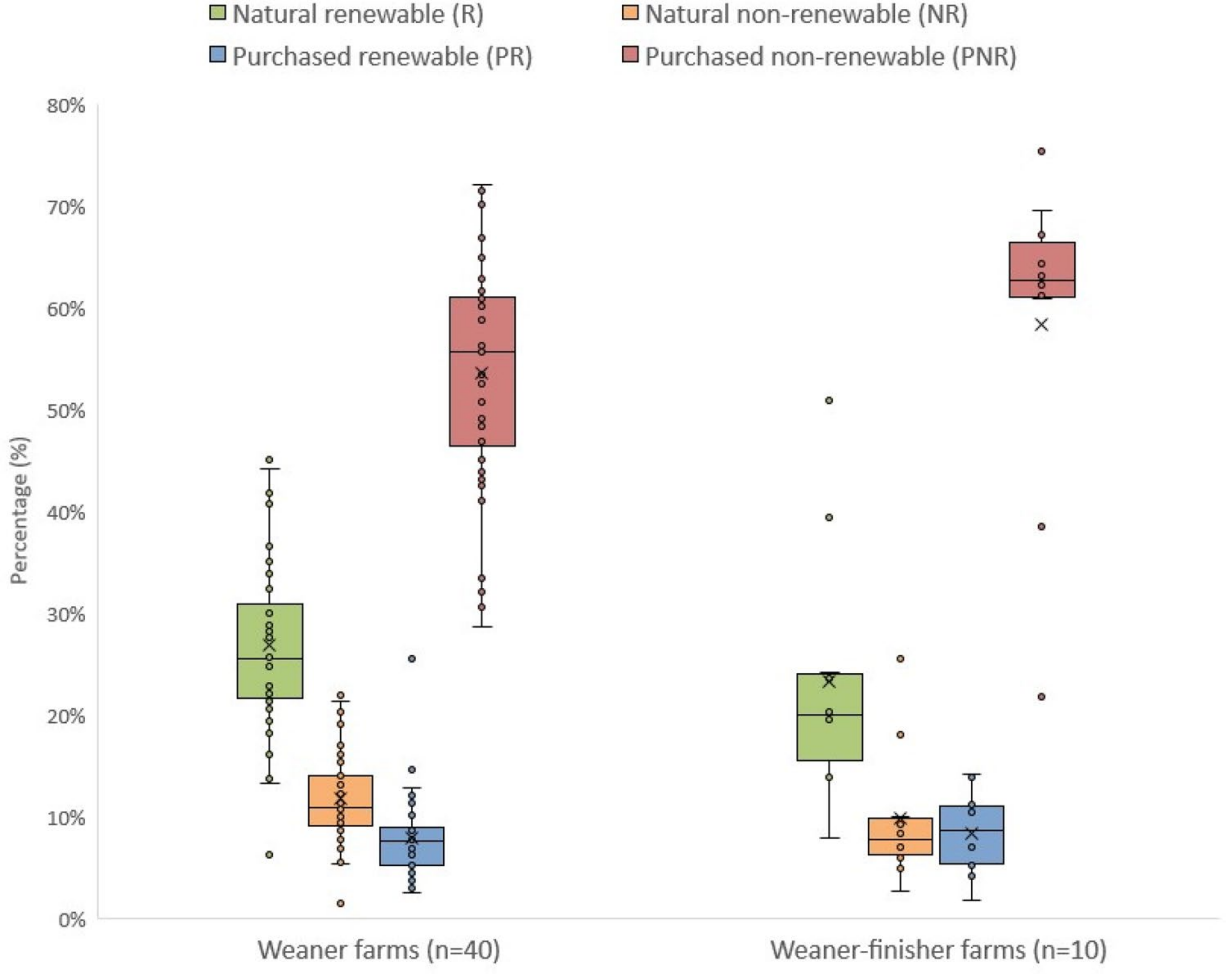
Distribution of the origin and renewability of the resources used by farming systems. Boxplots represent the farms (points), mean (crosses), median (solid horizontal lines), first and third quartiles (contained in boxes) and dispersion (vertical lines).

The proportion of Natural renewable local resources (R), Natural non-renewable resources (NR), Purchased renewable resources (PR) and Purchased non-renewable resources (PNR) across farms is provided in Fig. 3. For each farm, the sum of R, NR, PR and NPR is 100%. Heterogeneity among farms was wide, particularly on the weaner farms. The proportion of emergy from R varied between 6.3% and 50.9%, NR fluctuated between 1.5% and 25.5%, PR went from 1.8% to 25.4%, and PNR ranged from 21.8% to 75.4% of the total emergy flow.

### Emergy indicators—sustainability performance

**Figure 4:**
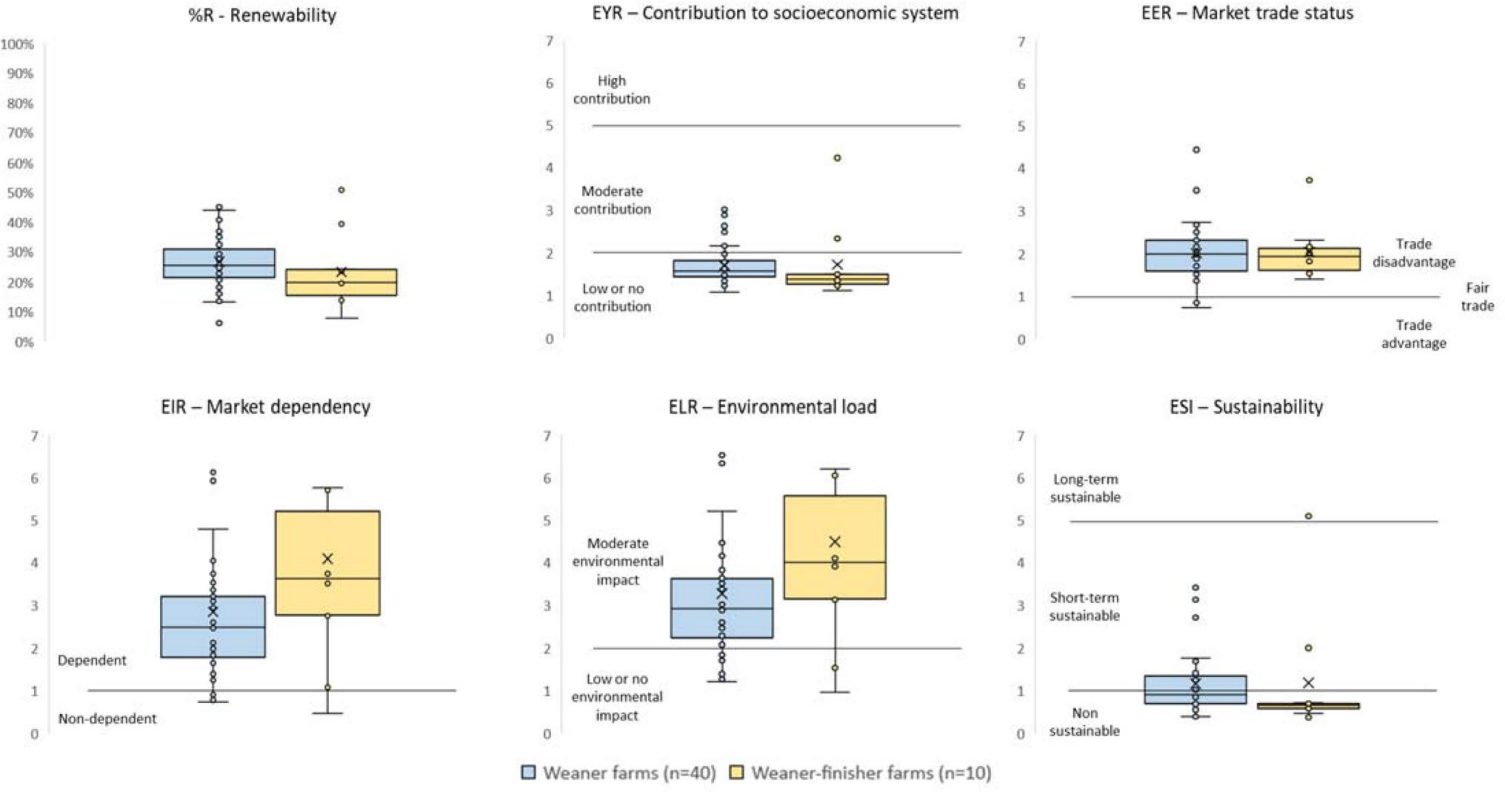
Farms’ environmental performance according to emergy indicators. Boxplots represent the mean (crosses), median (solid horizontal lines), first and third quartiles (contained in boxes), dispersion (boxes vertical lines) and outliers (external points) of the distribution of the indicators in farming systems. The thresholds of the emergy indicators were set^13,54^.

The results showed high heterogeneity across farms for most emergy indicators (Fig. 4). Despite this heterogeneity, there was a general pattern across farm in emergy performance. On average, around one fourth of the total emergy required to generate the final product came from renewable resources (%R) for both the weaner and weaner-finisher farms. Most farms did not incorporate significant net emergy into the socio-economic system (EYR ≤ 2) and depended on the market to maintain their activity (1 ≤ EIR). However, farms provided more emergy to the socio-economic system than what they received in return (1 < EER). Finally, farms had a variable, but moderate, environmental load (2 ≤ ELR). Because of their moderate environmental load (ELR) and minor contribution to the economy (EYR), around half the weaner farms were sustainable in the short term (1 ≤ ESI ≤ 5), while the other half and most of the weaner-finisher farms were unsustainable (ESI ≤ 1).

## Discussion

Demand for more sustainable agriculture and livestock production is increasing and more institutional efforts are being made in this regard^55^, notably for reducing GHG emissions at national and global levels^10,11^. Nonetheless, assessments that focus solely on GHG emissions commonly support farming systems that use less resources and emissions per product unit, regardless of the renewability of the used resources and, therefore, ignore whether the activity can be maintained in the long term or not^13^. Our study addresses livestock systems’ sustainability by offering a systemic and thermodynamic perspective that focuses on the energy and work required for nature to support the farming activity in a large farms sample.

Determining agricultural systems’ sustainability is a complex task that depends not only on efficiency and productivity, but also on the origin and renewability of the resources required and the load on the environment^44^. Despite pasture-based systems normally being considered to be highly sustainable because they depend on local renewable resources^8,56^, our analysis showed that, on average, only one fourth of farms’ emergy came from natural local renewable resources, which increased to one third when accounting for the renewable fraction of purchased resources. The high proportion of non-renewable emergy had a major driver in public economic support to farms (i.e., CAP payments received), whose marked contribution also resulted in an increase in the total emergy required to maintain farming activity. Consequently, most farms did not significantly contribute to the socio-economic system with net emergy (EYR ≤ 2), as has also been pointed out in other emergy assessments^31–33,57^ (see Supplementary material Table A2 for the comparative data of the emergy indicators). Farms showed moderate market dependence (EIR), which was greater than for similar farming systems^31–33,35,37,58^. Market dependence was driven mainly by animal feeds purchased and CAP payments received. These results are not surprising given European pasture-based systems’ low energy efficiency and their high economic dependence on public support^18–20^. Despite these farming systems poorly contributing to the socio-economic system (EYR), they are at a market trade disadvantage^54^ because they deliver more emergy to the socio-economic system than what they receive in return (EER > 1). In other words, emergy accounting brings a different perspective on the market exchange of pasture-based products: in economic terms they are receivers of public economic support, in biophysical terms they are donors of resources.

Regarding farming-environment interaction, our results showed that although there is variability across farms all of them present a moderate load on the environment (ELR > 2) due to the quite large inflow of non-renewable resources. The performance of weaner versus weaner-finisher farms cannot be compared because of their different product orientations. However, it seems that the more animal feeds purchased to fatten calves resulted in using more non-renewable resources and, therefore, depending more on the market, having higher environmental loads and worse sustainability performance. Consequently, only half the weaner farms and practically none of the weaner-finishers farms proved sustainable in the short term (1 < ESI > 5). We should note that farms are not far from being sustainable (in emergy terms), due to grazing and forage self-sufficiency. Therefore, increasing the use of grazing resources and self-produced forages could move farms towards sustainability. Our results revealed similar sustainability outputs to other cattle grazing systems^31,32,57^, but also lower than others^33,35,37,58^. There are two main reasons for the limited sustainability performance we found. The first reason is the strong economic dependence on public economic support^18^, which reduces sustainability by transferring the socio-economic system’s unsustainability (fossil fuels dependence) to farms^13^. The second reason is methodological and refers to the marked reduction in the emergy inflow from natural pastures according to our calculation that better captures the amount of emergy that goes into the system through livestock grazing (discussed below). Indeed, applying the standard calculation would have resulted in higher sustainability (moving from ESI≈1 to ESI > 3). In other words, most farming systems would have appeared as sustainable in the short-term.

An accurate accounting of grazing is crucial for sound sustainability assessments of pasture-based systems given the importance of local feed resources, particularly grazing in natural pastures beyond farm boundaries^42,59^. In quantitative terms, our calculation implied an average 75% reduction in the natural pastures emergy flow compared to the standard calculation and, accordingly, the inflow of natural renewable local emergy and farms’ sustainability declined. However, from a wider perspective, our calculation revealed the emergy from natural pastures that is not used by livestock, but maintains other ecosystem functions^53^. In qualitative terms, our calculation allowed us to recognise the value (in emergy terms) of ecosystems’ functions beyond their utility for human activities^60^. It may seem contradictory that natural pastures, which are a major source of animal feeding in pasture-based systems^18,43^, represent less than 4% of the emergy flow in these pasture-based livestock systems. However, it is precisely their low emergy contribution that makes natural pastures a key resource because, according to the emergy theory, the resources with the lowest transformities require less energy, work and intermediary transformations from the environment to be produced^13^.

Farm sustainability is not solely due to differences among farming systems (e.g., conventional, organic, low-input/low-output or pasture-based), but is also due to the relative importance of the resources they use, the farm structure and the specific farming practices (e.g., utilised agricultural area, herd size or purchased animal feeds) within the same farming systems^61^. In fact, farm sustainability greatly relies on farmers’ selection of farm resources, since the energy to produce those resources may come from solar radiation (constant but limited inflow) or fossil fuels, which can be used without limit but are a finite resource^60^. This means that despite the studied farms being managed under similar mountain conditions, the heterogeneity across farms in the relative importance of the used resources (natural renewable, natural non-renewable, purchased renewable and purchased non-renewable) is wide, which results in different sustainability performance across individual farms. Specifically, decisions at the farm level can reduce the unsustainable practices associated with soil erosion and improve feeding and grazing management^61,62^. However, even if farms could completely rely on self-produced feeds, avoid soil erosion, and leave out fertilisers and machinery, there would still be 54.7% and 48.3% (sum of evapotranspiration, services and labour for the weaner and weaner-finisher farms, respectively) of the total emergy that does not completely depend on farmers’ management decisions. Emergy accounting shows that farm sustainability is constrained not only by biophysical conditions, but also by the sustainability performance of the socio-economic system into which farms are integrated. The latter is modifiable only through changes made in society at large^13^, pointing to the need of a systemic change that goes far beyond individual farms^63^. Therefore, public economic support through CAP payments not only restrict economic sustainability, but also compromise environmental sustainability.

The quantification of dependence on non-renewable resources of farming systems, particularly pasture-based livestock systems, is especially relevant today, when the effects of climate and ecological crises call for reductions in fossil fuel use^64,65^. The depletion of these resources could cause energy deficits that threaten the viability of systems that strongly depend on fossil fuels^66^. Therefore, if policy goals and scientific recommendations for reducing fossil fuel consumption were followed^2^, the emergy from services and other purchased resources would be based on a higher proportion of renewable resources and, therefore, these pasture-based systems would become more sustainable. Our results also show that the repeatedly mentioned low economic viability of pasture-based systems is not related to biophysical barriers, but to the mainstream economic regime. This regime does not account for the free contribution of nature, but its time-specific price that depends on market fluctuations, resources scarcity and people’s willingness to pay^67^. Therefore, sustainability assessments such as emergy accounting can help to inform policymakers and avoid short-term measures, which are frequently driven by the ambition of continuous growth, and rely on the extended (and refuted) idea of fully decoupling economic growth and environmental impact^68,69^.

## Limitations

Emergy accounting has been largely developed in the last few decades^70^, and it is important to contemplate its shortcomings^71^ and to point out some weaknesses that call for the numerical results to be carefully read. First, the accuracy of the numerical emergy accounting results depends on the precision of the conversion factors (UEVs). This is particularly relevant when assessing specific goods production in local systems where specific UEVs are not normally available; in our case, some animal feeds like straw or forage are often purchased from nearby farmers, but we used standard UEVs from the literature. Second, this research relies on data from only 1 year. Therefore, annual monitoring could provide further insights to understand the impact of farmers decisions on farming sustainability. Third, there is some uncertainty when estimating the real pasture area used during the grazing season and the stocking rates. We reduced uncertainty by using available estimations for the study area. Fourth, mountain grazing livestock systems in the study area do not only produce food (meat), but also several ecosystem services recognised by society^72^. Despite emergy accounting allows ecosystem services to be evaluated and measured^73,74^, we did not have any empirical data. Thus, this study does not consider non-marketable services, which would have certainly improved sustainability outcomes.

## Conclusion

Our study broadens the view of mountain pasture-based systems’ sustainability by quantifying the origin, quality and quantity of the energy used across a group of farms. We assessed the relation of farming systems to the environment and the socio-economic system by showing the long-term feasibility of maintaining grazing systems.

Farms are strongly dependent on non-renewable resources due to the purchase of animal feeds and their dependence on the socio-economic system through CAP payments. This questions farms future viability. Farmers can improve the sustainability of their farms by increasing self-produced feeds and extending the grazing period length to maximise the use of renewable resources. However, the capacity of farms to improve sustainability is constrained by the functioning of the socio-economic system at large, which translates its energy unsustainability to mountain pasture-based farming. There is an urgent need to increase the sustainability of the global socio-economic system, which would determine the performance of the systems operating within its boundaries.

### Supplementary Information

Supplementary Information.

## Data Availability

The datasets generated and/or analysed during the current study are available in the citaREA repository, https://hdl.handle.net/10532/5863.
