# Supplementary material for: Dependence on the socio-economic system impairs the sustainability of pasture-based animal agriculture
Source: Sci Rep. 2023 Aug 31;13:14307. doi: 10.1038/s41598-023-41524-4 (PMC10471625; doi:10.1038/s41598-023-41524-4)
Supplement: Supplementary file 1 — Supplementary Information. [file 41598_2023_41524_MOESM1_ESM.docx]

**Dependence on the socio-economic system impairs the sustainability of pasture-based animal agriculture**

Muñoz-Ulecia, E.^1,2, *^, Bernués, A.^1,2^, Briones-Hidrovo, A.^3^, Casasús, I. ^1,2^, Martín-Collado, D. ^1,2^

*^1^ Department of Animal Science, Agrifood Research and Technology Centre of Aragon (CITA).* *Avda. Montañana 930, 50059 Zaragoza, Spain. ^2^ AgriFood Institute of Aragon – IA2 (CITA-University of Zaragoza), Zaragoza, Spain. ^3^ Research Centre for Energy Resources and Consumption (CIRCE)-University of Zaragoza-Campus Río Ebro, 50018 Zaragoza, Spain.*

**Corresponding author: emunnozul@cita-aragon.es*

Table A1. Description of the emergy variables calculation and the conversion factors (UEV) references.

| Variable | Items | Value | Units | Reference |
| --- | --- | --- | --- | --- |
| Sun radiation, J | (Area)·(Avg. Total annual Insolation)·(1-Albedo) | | |  |
|  | Area |  | m^2^ |  |
|  | Insolation | 5.63E+09 | J·m^-2^·yr^-1^ | Sistema de Información Agroclimática para el Regadío (SiAR) for 2018  http://eportal.miteco.gob.es/websiar/SeleccionParametrosMap.aspx?dst=2 |
|  | Albedo | 0.18 |  | NASA ASDC (https://asdc.larc.nasa.gov/sse/) |
|  | Annual energy |  | J·yr^-1^ |  |
|  | UEV | 1 | sej·J^-1^ | Odum, 1996 |
| Wind, J | (Area)·(Air density)·(Drag coefficient)·(Geostrophic wind velocity^3^) | | |  |
|  | Area |  | m^2^ |  |
|  | Air density | 1.24E+00 | kg·m^-3^ | Rodriguez-Ortega et al, 2017 following ideal gas law |
|  | Avg. annual wind velocity | 7.46E-01 | m·s^-1^ | Sistema de Información Agroclimática para el Regadío (SiAR) for 2018  http://eportal.miteco.gob.es/websiar/SeleccionParametrosMap.aspx?dst=2 |
|  | Geostrophic wind | 4.67E+00 | m·s^-1^ |  |
|  | Drag coefficient | 3.90E-01 |  | Rodriguez-Ortega et al, 2017 from Gillies et al, 2002 |
|  | Annual energy |  | J·yr^-1^ |  |
|  | UEV | 1.91E+03 | sej·J^-1^ | Odum, 2000 |
| Rain, J | (Area)·(Rainfall)·(Gibbs free energy of rainwater)·(1-runoff) | | |  |
|  | Area |  | m^2^ |  |
|  | Rainfall |  | m^3^·m^-3^·yr^-1^ |  |
|  | Gibbs free energy | 4.94 | J·g^-1^ |  |
|  | Conversion factor | 1.00E+06 | g·m^-3^ |  |
|  | Runoff coefficient | 3.40E-01 |  | Rodriguez-Ortega et al, 2017 from Prevert Table, 1986 |
|  | Annual energy |  | J·yr^-1^ |  |
|  | UEV | 4.08E+04 | sej·J^-1^ | Odum, 1996 |
| Evapotranspiration, J | (area)·(ETR)·(Gibbs free energy of rainwater) | | |  |
|  | Area |  | m^2^ |  |
|  | ETR | 9.12E-01 | m^3^·m^-3^·yr^-1^ | Sistema de Información Agroclimática para el Regadío (SiAR) for 2018 http://eportal.miteco.gob.es/websiar/SeleccionParametrosMap.aspx?dst=2 |
|  | Gibbs free energy | 4.94 | J·g^-1^ |  |
|  | Conversion factor | 1.00E+06 | g·m^-3^ |  |
|  | Annual energy | 6.75E+13 | J·yr^-1^ |  |
|  | UEV | 3.67E+04 | sej·J^-1^ | Campbell et al, 2005 |
| Soil erosion, J | (farmed area)·(erosion rate)·(%O.M. in soil)·(energy in O.M.) | | |  |
|  | Area |  | m^2^ |  |
|  | Erosion rate | 1201 | g·m^-2^·yr^-1^ | ICONA, Ministerio de Agricultura, Pesca y Alimentación |
|  | % Organic Matter (O.M.) in soil | 0.0195 |  | Rodriguez-Martín et al, 2009 |
|  | Energy in O.M. | 5.4 | kcal·g^-1^ |  |
|  | Conversion factor | 4186 | J·kcal^-1^ |  |
|  | Annual energy |  | J·yr^-1^ |  |
|  | UEV | 1.59E+05 | sej·J^-1^ | Odum, 1996 |
| Cow, weaned and fattened calves feed (commercial concentrates), g | Annual consumption | | |  |
|  | Annual consumption |  | g·yr^-1^ |  |
|  | UEV | 1.15E+09 | sej·g^-1^ | Vigne et al, 2013 |
| Minerals, g | Annual consumption | | |  |
|  | Annual consumption |  | g·yr^-1^ |  |
|  | UEV | 1.88E+09 | sej·g^-1^ | Vigne et al, 2013 from Odum, 2000 |
| Straw, g | Annual consumption | | |  |
|  | Annual consumption |  | g·yr^-1^ |  |
|  | UEV | 5.04E+08 | sej·g^-1^ | Guan et al, 2016 |
| Forage, J | Annual consumption | | |  |
|  | Annual consumption |  | g·yr^-1^ |  |
|  | UEV | 1.02E+05 | sej·J^-1^ | Ulgiati et al, 1994 |
| Corn, g | Annual consumption | | |  |
|  | Annual consumption |  | g·yr^-1^ |  |
|  | UEV | 1.51E+09 | sej·J^-1^ | Guan et al, 2016 |
| Seeds | Annual consumption | | |  |
|  | Annual consumption |  | J·yr^-1^ |  |
|  | UEV | 9.90E+04 | sej·J^-1^ | Ferreyra, 2001 |
| Fertilizers (nitrogen), g | Annual consumption | | |  |
|  | Annual consumption |  | g·yr^-1^ |  |
|  | UEV | 1.84E+10 | sej·g^-1^ | Brandt-Williams, 2002 |
| Phytochemical, g | Annual consumption | | |  |
|  | Annual consumption |  | g·yr^-1^ |  |
|  | UEV | 1.90E+10 | sej·g^-1^ | Brandt-Williams, 2002 |
| Veterinary services, € | Annual consumption | | |  |
|  | Annual consumption |  | €·yr^-1^ |  |
|  | UEV | 2.43E+12 | sej·€^-1^ | Own estimation from https://cep.ees.ufl.edu/nead/data.php# |
| Electricity, kWh | Annual consumption*Conversion factor | | |  |
|  | Annual consumption |  | kWh·yr^-1^ |  |
|  | Conversion factor | 3.6E+06 | J·kWh^-1^ |  |
|  | UEV | 5.37E+04 | sej·J^-1^ | Rodriguez-Ortega et al, 2017 |
| Fuels, J | Annual consumption | | |  |
|  | Annual consumption |  | J·yr^-1^ |  |
|  | UEV | 1.45E+05 | sej·J^-1^ | Campbell et al, 2005 |
| Small equipment, € | Annual consumption | | |  |
|  | Annual consumption |  | €·yr^-1^ |  |
|  | UEV | 2.43E+12 | sej·€^-1^ | Own estimation from https://cep.ees.ufl.edu/nead/data.php# |
| General maintenance (machinery, buildings), € | Annual consumption | | |  |
|  | Annual consumption |  | €·yr^-1^ |  |
|  | UEV | 2.43E+12 | sej·€^-1^ | Own estimation from https://cep.ees.ufl.edu/nead/data.php# |
| Amortization machinery, € | Annual consumption | | |  |
|  | Annual consumption |  | €·yr^-1^ |  |
|  | UEV | 2.43E+12 | sej·€^-1^ | Own estimation from https://cep.ees.ufl.edu/nead/data.php# |
| Amortization buildings, € | Annual consumption | | |  |
|  | Annual consumption |  | €·yr^-1^ |  |
|  | UEV | 2.43E+12 | sej·€^-1^ | Own estimation from https://cep.ees.ufl.edu/nead/data.php# |
| Human labour, J | (pers-hour·yr^-1^)·(kcal pers·day^-1^)/(8 pers-hour·day^-1^) | | |  |
|  | pers-hour |  | pers-hour |  |
|  | kcal pers·day^-1^ | 2500 | kcal |  |
|  | Conversion factor | 4186 | J·cal^-1^ |  |
|  | UEV | 3.40E+06 | sej·J^-1^ | Brandt-Williams, 2002 |
| Taxes, € | Annual cost | | |  |
|  | Annual cost |  | €·yr^-1^ |  |
|  | UEV | 2.43E+12 | sej·€^-1^ | Own estimation from https://cep.ees.ufl.edu/nead/data.php# |
| CAP payments, € | Annual cost | | |  |
|  | Annual cost |  | €·yr^-1^ |  |
|  | UEV | 2.43E+12 | sej·€^-1^ | Own estimation from https://cep.ees.ufl.edu/nead/data.php# |

The real farm values for annual consumption/production for the 50 observations were used for the calculation.

**References for Table A1.**

Brandt-Williams, S. L. Folio# 4. Emergy of Florida Agriculture. *Handbook of Emergy Evaluation: A Compendium of Data for Emergy Computation Issued in a Series of Folios*, 40 (2002).

Campbell, D. E., Brandt-Williams, S. L., & Meisch, M. E. Environmental accounting using emergy: Evaluation of the state of West Virginia. *US Environmental Protection Agency, Office of Research and Development, National Health and Environmental Effects Research Laboratory, Atlantic Ecology Division* (2005).

Ferreyra, M. C. Emergy perspectives on the argentine economy and food production systems of the rolling pampas during the twentieth century (*Doctoral dissertation, University of Florida*) (2001).

Guan, F. *et al.* Emergy assessment of three home courtyard agriculture production systems in Tibet Autonomous Region, China. *J. Zhejiang Univ. Sci. B* 17, 628–639 (2016).

NASA Atmospheric Science Data Center. Available at <https://asdc.larc.nasa.gov/project/SSE>

National Environmental Accounting Database Center for Environmental Policy, University of Florida. Available at [https://cep.ees.ufl.edu/nead/data.php#](https://cep.ees.ufl.edu/nead/data.php).

Odum, H.T. Environmental accounting: emergy and environmental decision making. Wiley (1996).

Odum, H.T. Folio #2 Emergy global processes. *Handbook of Emergy Evaluation. A Compendium of Data for Emergy Computation. Center of Environmental Policy, University of Florida, Gainesville* (2000).

Rodríguez-Martín, J.A., López Arias, M., Grau Corbí, J.M. Metales Pesados, Materia Organica y Otros Parametros de los Suelos Agricolas y de Pastos de España*. Ministerio de medio ambiente y medio rural y marino/Instituto Nacional de Investigación y Tecnología Agraria y Alimentaria*, Madrid (2009).

Rodríguez-Ortega, T., Bernués, A., Olaizola, A. M., & Brown, M. T. Does intensification result in higher efficiency and sustainability? An emergy analysis of Mediterranean sheep-crop farming systems. *Journal of Cleaner Production*, 144, 171-179 (2017).

Sistema de Información Agroclimática para el Regadío (SiAR) for 2018. Available at http://eportal.miteco.gob.es/websiar/SeleccionParametrosMap.aspx?dst=

Ulgiati, S., Odum, H. T., & Bastianoni, S. Emergy use, environmental loading and sustainability an emergy analysis of Italy. *Ecological modelling*, 73(3-4), 215-268 (1994).

Vigne, M. *et al*. Emergy evaluation of contrasting dairy systems at multiple levels. *Journal of environmental management*, 129, 44-53 (2013).

Table A2. Summary table of the emergy indicators and comparison to similar studies. The average and standard deviation for our study.

| Emergy indicators | This study | | Alfaro-Arguello et al. (2010) | | dos Reis et al. (2021) | | Zhang et al. (2007) | Haden (2002) | Bastianoni et al. (2001) | Rótolo et al. (2007) | Fonseca et al. (2019, 2016) |
| --- | --- | --- | --- | --- | --- | --- | --- | --- | --- | --- | --- |
| System | Weaner farms (n=40) | Weaner-finisher farms (n=10) | Conventional (n=18) | Holistic (n=7) | Livestock subsystem (n=1) | Crop-livestock (n=1) | Crop-livestock ^a^ | Livestock subsystem (n=1) | Crop-livestock (n=1) | Grazing (n=1) | Grazing cattle subsystem (n=1) |
| Renewability (%R) (%) | 26.8 ± 8.5 | 23.3 ± 12.8 | - | - | 66.0 | 31.0 | 40.0 | - | - | - | 35.0 |
| Contribution to society (EYR) | 1.7 ± 0.4 | 1.7 ± 0.9 | 1.6 | 2.0 | 3.0 | 1.5 | 4.7 | 2.1 | 2.0 | 3.7 | 1.7 |
| Market dependency (EIR) | 2.8 ± 2.3 | 4.1 ± 3.1 | - | - | 0.5 | 2.1 | 0.3 | 0.9 | - | 0.4 | 1.5 |
| Trade status (EER) | 2.1 ± 0.6 | 1.9 ± 0.7 | - | - | - | - | - | - | - | - | 0.4 |
| Env. load (ELR) | 3.3 ± 2.2 | 4.5 ± 3.0 | 2.6 | 1.8 | 0.5 | 2.2 | 1.5 | 0.9 | 1.1 | 0.6 | 1.5 |
| Env. sustainability (ESI) | 1.1 ± 0.7 | 1.2 ± 1.5 | 1.0 | 1.6 | 5.6 | 0.7 | 3.1 | 2.2 | - | 6.8 | 1.1 |

The shown indicators are Renewability (%R), Emergy Yield Ratio (EYR), Emergy Investment Ratio (EIR), Emergy Exchange Ratio (EER), Environmental Loading Ratio (ELR), Emergy Sustainability Index (ESI). ^a^ Data from an entire region.
